# OPIOID DISCONTINUATION RATES: RISK STRATIFICATION USING A CLAIMS-BASED FRAILTY INDEX (2013–2018 MEDICARE)

**DOI:** 10.1093/geroni/igae098.1264

**Published:** 2024-12-31

**Authors:** Kevin Pritchard, Chun-Ting Yang, Qiaoxi Chen, Yichi Zhang, James Wilkins, Dae Hyun Kim, Kueiyu Joshua Lin

**Affiliations:** Brigham and Women’s Hospital, Boston, Massachusetts, United States; Brigham and Women’s Hospital, Boston, Massachusetts, United States; Brigham and Women’s Hospital, Boston, Massachusetts, United States; Brigham and Women’s Hospital, Boston, Massachusetts, United States; Harvard Medical School, Boston, Massachusetts, United States; Hebrew SeniorLife, Boston, Massachusetts, United States; Brigham and Women’s Hospital, Boston, Massachusetts, United States

## Abstract

We aimed to determine if the severity of frailty influences the duration of opioid use following an emergent pain event. This retrospective cohort study included 21,728 fee-for-service Medicare beneficiaries (2013-2018). We included opioid-naïve individuals aged ≥65 years who initiated opioids within 30 days of a hospitalized fracture. Our index date was the opioid prescription fill date. We excluded those with prior opioid use, chronic pain, or contraindications to opioids in the preceding year. Frailty severity was defined using a claims-based index. We estimated opioid discontinuation rates using Nelson-Aalen cumulative hazard functions, stratified by frailty severity. Discontinuation during the follow-up year was defined as a gap in continuous prescription refills exceeding 15 days. We used Cox proportional hazards regression to assess the association between frailty severity and the number of days until discontinuing opioids while controlling for sociodemographic factors, health status, healthcare utilization, and calendar year. Our sample was on average age 80 [SD=9] years old, 77% female, and 91% White. The 30-day discontinuation rate was 85% (95% CI, 83-87%) in the moderate/severe frail group, 84% (83%-84%) in the mildly frail group, 87% (87%-88%) in the pre-frail group, and 90% (89-91%) in the non-frail group. After multivariable adjustment, the likelihood to discontinue opioids depended on frailty status. Compared to adults without frailty, discontinuation was less likely among those who were moderately/severely frail (Hazard Ratio [HR]: 0.88, 95% CI, 0.83-0.94), mildly frail (HR: 0.87, 95% CI, 0.83-0.92), and pre-frail (HR: 0.96, 95% CI, 0.91-1.00). Greater frailty decreased the likelihood of discontinuing opioids.

